# Exercise and Training Regulation of Autophagy Markers in Human and Rat Skeletal Muscle

**DOI:** 10.3390/ijms23052619

**Published:** 2022-02-27

**Authors:** Javier Botella, Nicholas A. Jamnick, Cesare Granata, Amanda J. Genders, Enrico Perri, Tamim Jabar, Andrew Garnham, Michael Lazarou, David J. Bishop

**Affiliations:** 1 Institute for Health and Sport (iHeS), Victoria University, Melbourne, VIC 3011, Australia; n.jamnick@deakin.edu.au (N.A.J.); cesare.granata@monash.edu (C.G.); amanda.genders@vu.edu.au (A.J.G.); enrico.perri@unimi.it (E.P.); tamimjabar098@hotmail.com (T.J.); andrew.garnham@deakin.edu.au (A.G.); 2 Metabolic Research Unit, Institute for Mental and Physical Health and Clinical Translation (IMPACT), School of Medicine, Deakin University, Waurn Ponds, VIC 3011, Australia; 3 Department of Diabetes, Central Clinical School, Monash University, Melbourne, VIC 3011, Australia; 4 Department of Biomedical Sciences for Health, University of Milan, 20122 Milan, Italy; 5 Department of Biochemistry and Molecular Biology, Biomedicine Discovery Institute, Monash University, Melbourne, VIC 3011, Australia; michael.lazarou@monash.edu

**Keywords:** autophagy, exercise, LC3, skeletal muscle

## Abstract

Autophagy is a key intracellular mechanism by which cells degrade old or dysfunctional proteins and organelles. In skeletal muscle, evidence suggests that exercise increases autophagosome content and autophagy flux. However, the exercise-induced response seems to differ between rodents and humans, and little is known about how different exercise prescription parameters may affect these results. The present study utilised skeletal muscle samples obtained from four different experimental studies using rats and humans. Here, we show that, following exercise, in the soleus muscle of Wistar rats, there is an increase in LC3B-I protein levels immediately after exercise (+109%), and a subsequent increase in LC3B-II protein levels 3 h into the recovery (+97%), despite no change in *Map1lc3b* mRNA levels. Conversely, in human skeletal muscle, there is an immediate exercise-induced decrease in LC3B-II protein levels (−24%), independent of whether exercise is performed below or above the maximal lactate steady state, which returns to baseline 3.5 h following recovery, while no change in LC3B-I protein levels or *MAP1LC3B* mRNA levels is observed. *SQSTM1*/p62 protein and mRNA levels did not change in either rats or humans following exercise. By employing an ex vivo autophagy flux assay previously used in rodents we demonstrate that the exercise-induced decrease in LC3B-II protein levels in humans does not reflect a decreased autophagy flux. Instead, effect size analyses suggest a modest-to-large increase in autophagy flux following exercise that lasts up to 24 h. Our findings suggest that exercise-induced changes in autophagosome content markers differ between rodents and humans, and that exercise-induced decreases in LC3B-II protein levels do not reflect autophagy flux level.

## 1. Introduction

Autophagy is the cellular process by which an autophagosome (a double-membrane vesicle) engulfs, and delivers to the lysosome, proteins or organelles that need to be degraded. It is the recycling machinery of the cell and is important for the correct removal of intracellular pathogens or misfolded proteins, among others, which may activate deleterious cellular signalling pathways (e.g., inflammation) [1]. In skeletal muscle, autophagy is important to prevent mitochondrial damage [2], to promote positive muscle regeneration [3] and optimal glucose metabolism [4], and for training-induced increases in mitochondrial proteins and endurance performance [5]. Thus, it is important to better understand factors that influence autophagy in skeletal muscle.

The autophagy machinery consists of a core set of autophagy-related (ATG) proteins [6]. Among these, the ATG8 family (which includes the subfamily members LC3A, LC3B, LC3C, GABARAP, GABARAPL1, and GABARAPL2) promotes autophagosome formation and autophagosome–lysosome fusion [7,8]. In skeletal muscle, LC3s are abundantly expressed [9], which makes LC3 a widely used marker of autophagosome content. In mice, a single session of endurance exercise increased the level of LC3-II (and the LC3-II/I ratio), as well as the appearance of LC3 puncta in both skeletal and cardiac muscle [4], and exercise to exhaustion increased the protein levels of LC3-II and the autophagy receptor p62 in the tibialis anterior (TA), along with a tendency for increased exercise-induced autophagy flux [10]. Following a similar exercise session in mice, LC3-I protein levels have also been reported to increase [11]. A study comparing two different exercise regimes in mice showed that both low- and moderate-intensity exercise increased LC3A/B-II protein levels and the LC3A/B-II/I ratio 3 h following the end of exercise [12]. Similarly, in rats, a single session of endurance exercise increased the LC3B-II protein content and the LC3B-II/I ratio in the TA muscle [13]. Although findings are inconclusive regarding exercise-induced SQSTM1/p62 protein changes, the LC3 findings collectively suggest that, in rodents, autophagosome content, and possibly autophagy flux, are increased in a variety of skeletal muscles after a session of endurance exercise.

In contrast to rodent studies, human studies show a distinct pattern of exercise-induced changes in autophagosome content markers. Protein levels of LC3B-II and the LC3B-II/I ratio have been shown to decrease 0 to 1 h following different types of endurance exercise and return to baseline values after 3 to 4 h of recovery in human skeletal muscle [13,14,15,16]. In contrast, 60 min of exercise at 60% of $\dot{V}$O_2max_ has been reported to increase the levels of LC3A/B-II protein levels 2 h after the end of exercise [17]. Following most types of endurance exercise, the protein levels of the autophagy receptor SQSTM1/p62 remained unchanged [13,14,16,17]. In contrast, following 2 h at 70% of $\dot{V}$O_2peak_, but not at 55% of $\dot{V}$O_2peak_, the SQSTM1/p62 protein levels decreased, which could suggest an effect of exercise intensity on the exercise-induced SQSTM1/p62 protein changes [15]. However, since both the exercise intensity and the total work completed were different, it is difficult to isolate any of these factors. Other differences, such as training status, timing of biopsies, antibodies used, or sample size, may also have contributed to the reported discrepancies between human studies. Whether exercise intensity distinctly affects the LC3B and SQSTM1/p62 protein levels in humans following an exercise session requires further investigation.

Rodent studies have also shown that exercise training increased levels of autophagy proteins. Four weeks of voluntary wheel running exercise led to increases in LC3-II protein content of both the plantaris and soleus muscle of mice, while SQSTM1/p62 protein content was only increased in the oxidative soleus muscle [5]. Similarly, five weeks of exercise training (twice per day) increased LC3A/B-II protein and LC3A/B-II/I ratio in the quadriceps muscle of mice, with no change in SQSTM1/p62 protein content [12]. In aged rats, 12 weeks of treadmill or voluntary training led to increases in the LC3-II/I ratio [18]. While the majority of rodent studies suggest that LC3-II protein content increases following exercise training, the findings in humans remain uncertain. Eight weeks of endurance exercise showed that only LC3A/B-I, but not LC3A/B-II, protein content changed [17]. Conversely, a recent study showed that intensifying the training of elite endurance athletes, by adding three extra exercise sessions per week for four weeks, increased the protein content of LC3B-II [19]. Future studies should explore whether exercise training volume is important for training-induced changes in markers of autophagy.

Autophagy flux assays are considered the ‘gold-standard’ to assess autophagy levels [20]. Autophagy flux is the term used for the combined autophagy steps, which includes autophagosome formation, maturation, fusion with lysosomes, and breakdown of the autolysosome contents. One such assay aims to chemically block the fusion of autophagosomes with the lysosome (the end-point of the degradation process) and to monitor the accumulation of LC3-II [20]. Performing an in vivo autophagy flux is not ethically possible in human tissues and remains a limitation. This means that human studies have relied on markers of autophagosome and autophagy receptor protein levels [13,14,15,16,17]. Although not previously used in humans, animal models have also utilised an ex vivo autophagy flux analysis [21]. Implementing this ex vivo LC3-II flux assay could provide a direct assessment of autophagy in human studies and would avoid having to rely solely on indirect markers (i.e., LC3-II/I ratio). 

Despite the increase in autophagy research in skeletal muscle, there is currently no consensus on the exercise-induced regulation of autophagosome content in skeletal muscle. The aims of the current study are multiple: (1) to assess potential differences in exercise-induced changes in LC3B and SQSTM1/p62 protein between rodents and humans; (2) to elucidate if the exercise-induced LC3B and SQSTM1/p62 protein changes are affected by exercise below or above the maximal lactate steady state (MLSS) in humans; (3) to explore the effects of exercise training on the basal LC3B and SQSTM1/p62 protein levels in humans; and (4) to provide a preliminary assessment of whether the acute exercise-induced changes in LC3B-II protein levels are reflective of a decreased ex vivo autophagy flux in humans.

## 2. Results

### 2.1. Study 1—Exercise-Induced Changes in LC3B and SQSTM1/p62 Gene and Protein Changes in the Soleus Muscle of Male Wistar Rats

**Figure 1:**
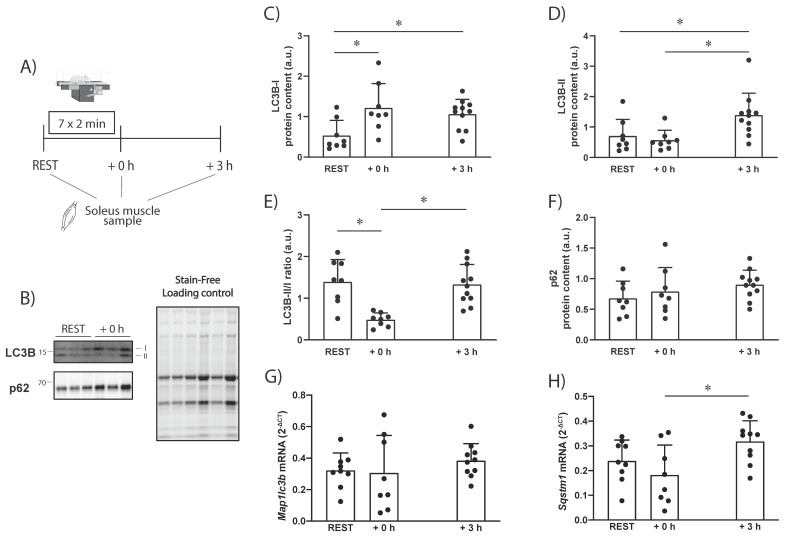
(**A**) Schematic representation of the study design and timing of sample collection. (**B**) Representative blots of LC3B, p62, and total protein using a stain-free system. (**C**) Effects of a single session of high-intensity interval exercise (7 × 2 min) on LC3B-I and (**D**) LC3B-II protein levels; (**E**) the LC3B-II/I ratio and (**F**) p62 protein levels in the soleus muscle of Wistar rats; (**G**) mRNA levels of *Map1lc3b*, the gene encoding LC3B; (**H**) mRNA levels of *Sqstm1*, the gene encoding p62. * = *p* < 0.05. Bars are shown as mean + SD.

There was a main effect of time for LC3B-I and LC3B-II protein levels (both *p* = 0.01), as well as the LC3B-II/I ratio (*p* = 0.0003). Compared to REST, there was a significant increase in LC3B-I protein level at +0 h (+109 ± 103%; ES = 1.1; *p* < 0.0001; Figure 1C) and at +3 h (+82 ± 62%; ES = 1.1; *p* < 0.0001; Figure 1C). Compared to REST, LC3B-II protein level did not significantly change at +0 h (−20 ± 46%; ES = −0.32; *p* = 0.63; Figure 1D), but significantly increased at +3 h (+97 ± 102%; ES = 0.95; *p* = 0.04; Figure 1D), and from +0 h to +3 h (+159 ± 129%; ES = 1.2; *p* = 0.02; Figure 1D). There was a significant decrease in the LC3B-II/I ratio immediately (+0 h) after exercise (−65 ± 12%; ES = −1.5; *p* = 0.001; Figure 1E), followed by a significant increase from +0 h to +3 h (+164 ± 98%; ES = 1.5; *p* = 0.001; Figure 1E). The protein level of SQSTM1/p62 did not significantly change at any time point (*p* > 0.05; Figure 1F). The mRNA levels of *Sqstm1* were only significantly changed from +0 h to +3 h (+74 ± 45%; ES = 1.12; *p* = 0.02; Figure 1H). There were no significant differences between time points for mRNA levels of *Map1lc3b*.

### 2.2. Study 2—Effects of Exercise Intensity on Exercise-Induced LC3B and SQSTM1/p62 Gene and Protein Changes in Male Human Skeletal Muscle

**Figure 2:**
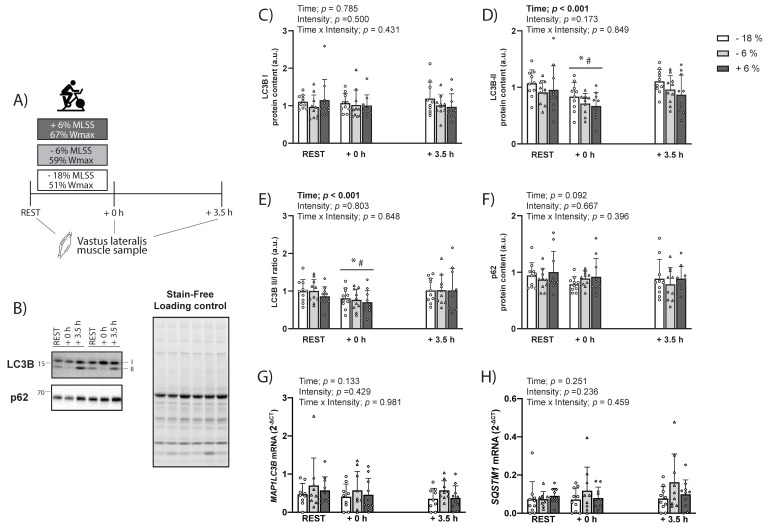
(**A**) Schematic representation of the study design and timing of sample collection. Participants completed the exercise at three different intensities (−18% = white, −6% = grey, and +6% = black, of the individually determined maximal lactate steady state (MLSS); n = 10). (**B**) Representative blots of LC3B, p62, and total protein using a stain-free system. (**C**) Protein levels of LC3B-I and (**D**) LC3B-II at rest, as well as 0 h and 3.5 h after the end of exercise; (**E**) the LC3-BII/I ratio and (**F**) p62 protein levels at rest, 0 h, and 3.5 h after the end of exercise; (**G**) mRNA levels of MAP1LC3B, the gene encoding LC3B; (**H**) mRNA levels of SQSTM1, the gene encoding p62. * = significantly different from REST (*p* < 0.05); # = significantly different from +3.5 h (*p* < 0.05). Bars shown are mean + SD.

There was no main or interaction effect for LC3B-I protein levels (*p* > 0.05; Figure 2C). There was no time x intensity, or intensity, effect for LC3B-II protein levels (*p* < 0.05), but there was a main effect of time (*p* < 0.0001). Compared to REST, there was a significant decrease at +0 h (−24 ± 16%; 90% CI (−28, −19%); ES = −0.82; *p* = 0.0001; Figure 2D), but not at +3 h (+6 ± 31%; 90% CI (−4, 15%); ES = 0.01; *p* > 0.99; Figure 2D), and a significant increase between +0 h and +3 h (+40 ± 38%; 90% CI (28, 51%); ES = 0.85; *p* < 0.0001; Figure 2D). There was no time x intensity, or intensity, effect for LC3B-II/I ratio (*p* > 0.05), but there was a main effect of time (*p* < 0.0001). Compared to REST, there was a significant decrease at +0 h (−21 ± 17%; 90% CI (−26, −16%); ES = −0.65; *p* = 0.003; Figure 2E), but no significant difference at +3 h (+10 ± 45%; 90% CI (−4, 24%); ES = 0.18; *p* = 0.27; Figure 2E), and a significant difference between +0 h and +3 h (+40 ± 45%; 90% CI (27, 54%); ES = 0.67; *p* = 0.0001; Figure 2E). There was no main or interaction effect for SQSTM1/p62 protein levels (all *p* > 0.05; Figure 2F). There were no significant effects or interactions in *SQSTM1* or *MAP1LC3B* mRNA levels (all *p* > 0.05; Figure 2G,H).

### 2.3. Study 3—Effects of High-Volume HIIT on Resting LC3B and SQSTM1/p62 Protein Levels in Male Human Skeletal Muscle

**Figure 3:**
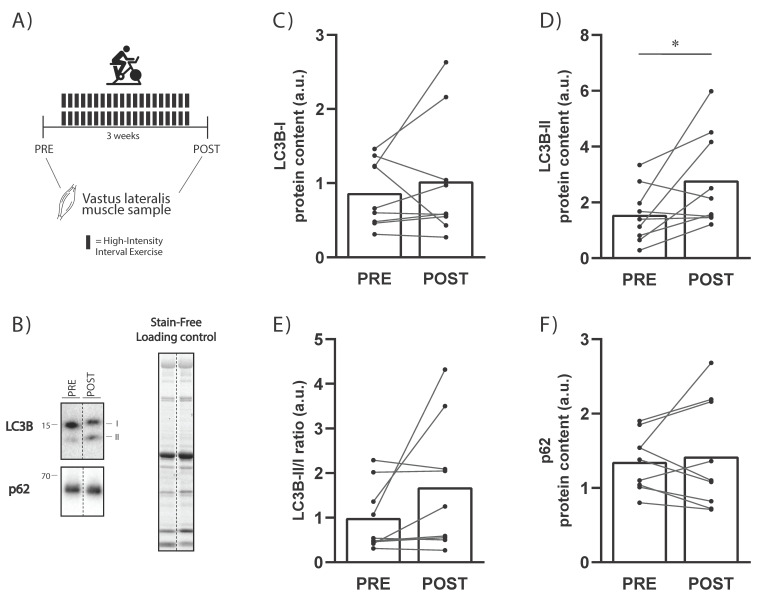
(**A**) Schematic representation of the training study design and timing of sample collection. (**B**) Representative blots of LC3B, p62, and total protein using a stain-free system. (**C**) Protein levels of LC3B-I and (**D**) LC3B-II in PRE and POST training samples; (**E**) the LC3B-II/I ratio and (**F**) p62 protein levels in PRE and POST training samples. * = significantly different from PRE training sample (*p* < 0.05). Individual and mean changes are shown.

Resting levels of LC3B-II protein levels significantly increased from PRE to POST (+132 ± 140%; 90% CI (+55, 209%); ES = 0.85; *p* = 0.04; Figure 3D). There was no significant training effect on LC3B-I, LC3B-II/I ratio, or SQSTM1/p62 protein levels (all *p* > 0.05; Figure 3).

### 2.4. Study 4—Exercise-Induced Changes in Autophagy Flux in Male Human Skeletal Muscle

**Figure 4:**
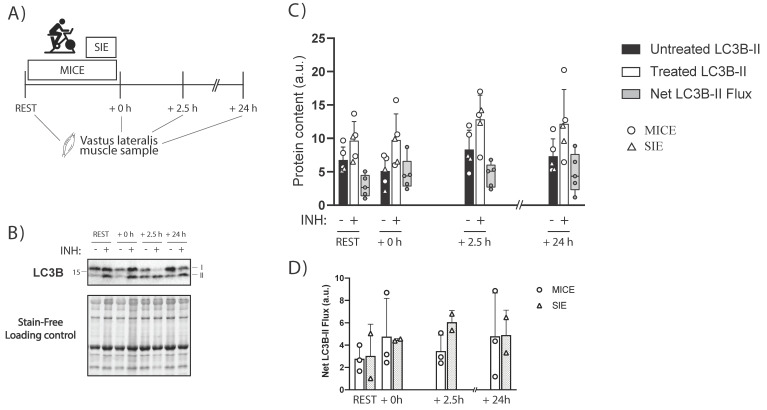
(**A**) Schematic representation of the study design and timing of sample collection. (**B**) Representative blot of LC3B and total protein using a stain-free system. (**C**) LC3B-II protein levels from untreated (black bars) and treated samples (white bars), and the net LC3B-II flux (in grey; calculated by subtracting untreated LC3B-II protein levels from treated sample). (**D**) Net LC3B-II flux divided by exercise groups. Triangles represent participants performing sprint-interval exercise (SIE; n = 2) and circles represent participants performing moderate-intensity continuous exercise (MICE; n = 3). Bars for the treated and untreated samples display the mean + SD. Individual data points, along with box and whisker plots, are shown for net LC3B-II flux. INH = inhibitors NH_4_Cl (40 mM) and Leupeptin (100 µM) were added to the treated sample.

There was a main effect for LC3B-II protein levels in untreated samples (*p* = 0.017; Figure 4). Compared to REST there were no significant changes at +0 h (−26 ± 23%; 90% CI (−43, −10%); ES = −0.77; *p* = 0.10), at +2.5 h (+23 ± 27%; 90% CI (3, 43%); ES = 0.64; *p* = 0.24), or at +24 h (+8.7 ± 23%; 90% CI (−8, 26%); ES = 0.26; *p* = 0.53). However, relative changes from REST to +0 h between the samples from Study 2 and those in Study 4 were comparable (−26% vs. −24% respectively; and ES = −0.77 vs. −0.82, respectively), suggesting a similar exercise-induced response in LC3B-II across experiments in the untreated samples.

For net LC3B-II flux, there was no main effect (*p* = 0.27). Compared to REST, effect size analyses showed a large positive change at +0 h (+117 ± 163%; 90% CI (−3, 237%); ES = 0.82; *p* = 0.22), at +2.5 h (+113 ± 178%; 90% CI (−18, 244%); ES = 0.88; *p* = 0.22), and at +24 h (+93 ± 126%; 90% CI (1, 126%); ES = 0.79; *p* = 0.22).

## 3. Discussion

Our study shows that: (1) exercise-induced changes in LC3B protein levels differ between rats and humans; (2) exercise-induced changes in LC3B and SQSTM1/p62 protein levels appear to be independent of exercising at an intensity below or above the MLSS in human skeletal muscle; (3) LC3B-II protein level increases following a high-intensity high-volume training period in humans; and (4) the exercise-induced decrease in LC3B-II protein levels observed in humans were not reflective of a decrease in autophagy flux. 

The results of the present study showed that the exercise-induced changes in LC3B protein levels differ between rats and humans. In our rat study, there were increased LC3B-I protein levels 0 to 3 h following a single endurance exercise session (Figure 1). This was not observed in our human study (Figure 2), in accordance with previous literature [13]. Our findings did not show a change in *Map1lc3b* mRNA, suggesting increased translation and/or reduced protein breakdown may have contributed to the observed increase in LC3B-I protein in rats. Contrary to our results, M*ap1lc3b* mRNA expression has been reported to increase following exercise to exhaustion in mice [10], which coincides with an exercise-induced increase in LC3-I protein levels that has also been shown in mice [11]. In humans, LC3B gene expression is not generally increased following endurance exercise [22], and no endurance-exercise-induced increase in LC3B-I protein level has been reported. However, it is difficult to compare across studies, as not many studies report the changes in LC3-I protein levels.

**Figure 5:**
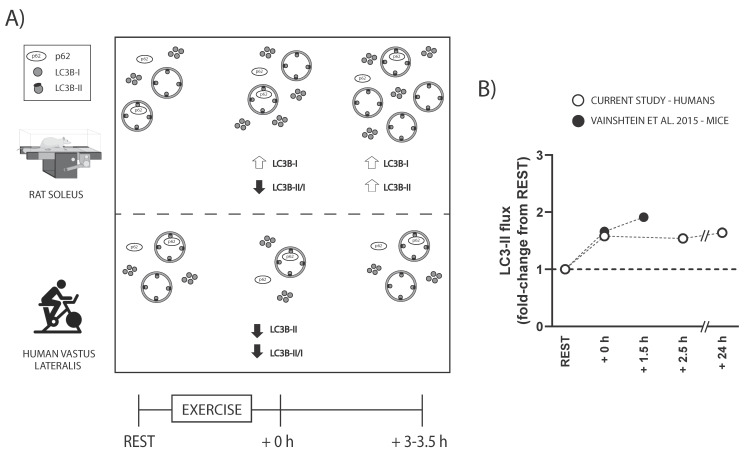
(**A**) Summary and model of the findings of the present study combining the multiple exercise studies. (**B**) Illustrative comparison of the exercise-induced changes in LC3-II flux from the present study (LC3B-II, human) and a previously published mice study (LC3-II, adapted from [10]).

In the present study, LC3B-II protein levels were unaltered immediately after exercise in rats but were significantly increased 3 h into the recovery (Figure 5A). This is in line with previous research showing that LC3-II is significantly increased in rodents 80 to 180 min from the start of exercise [4,13,23]. Due to the incomplete information regarding the antibodies used, it was not possible to recapitulate the findings for the LC3 subfamily members used in some of the rodent studies. Future research should address whether the different LC3 subfamily members are similarly modified following exercise in rodents. Our results show that the protein levels of the autophagy receptor SQSTM1/p62 remained unchanged at all time points in rats. While this is in contrast to some rodent studies [4,24], it is in agreement with most findings [10,11,12,25]. A possible explanation may relate to the duration of the exercise in the different studies, as the only two studies reporting an exercise-induced decrease in SQSTM1/p62 protein levels included exercise protocols lasting at least 110 min [4,24], and a decrease in SQSTM1/p62 was not seen at earlier time points or in the recovery period [24]. On the other hand, SQSTM1/p62 protein level has previously been shown to be decreased 6 h into the recovery from both low- and moderate-intensity exercise [12], suggesting a delayed lysosomal degradation of autophagosomes, which may have been missed by most studies, including the present study. It is important to mention that other proteins can also act as autophagy receptors (e.g., NBR1, OPTN [26]), and how these are altered by exercise requires further investigation.

In contrast to rodents, the findings from our human study show that, independently of exercising below or above the MLSS, LC3B-II protein levels were decreased following exercise and returned to baseline 3.5 h into the recovery (Figure 2 and Figure 5A). The results of study 2, therefore, suggest that exercise intensity may not be a factor determining changes in LC3B or SQSTM1/p62 protein and mRNA levels under our experimental conditions. While this is in contrast with a previous study [15], their design did not control for total work, as both exercise intensity and volume differed between their groups. Therefore, we suggest that total volume of exercise, or a combination of a high volume of exercise with increased intensity, rather than exercise intensity per se, may be more important to elicit different autophagy-related responses. Our results of an acute exercise-induced decrease in LC3B-II protein were in accordance with most human studies [13,14,15,16], with one exception [17]. A major difference with the study of Brandt, Gunnarsson [17] was the protein analysed. In contrast to the present study and others where an antibody targeting the LC3B subfamily was utilised [13,14,15,16], Brandt, Gunnarsson [17] used an antibody targeting a combination of LC3A and LC3B. Whether the protein levels of the different LC3 subfamily members are differentially regulated following exercise remains to be elucidated. Interestingly, a proteomic analysis of human skeletal muscle studies only detected LC3A [27], which may indicate a greater protein abundance of LC3A when compared to the other subfamily members in skeletal muscle. The present findings also show that LC3B-I protein levels were not altered following exercise, consistent with previous studies in humans [14,16]. The unchanged LC3B-I protein levels could be due to unchanged mRNA expression of LC3B or rapid conjugation of LC3-I into LC3-II and increased autophagosome degradation. Our results did not show a change in the mRNA levels of *MAP1LC3B* as a potential mechanism controlling the unchanged LC3B-I protein content. The finding of unchanged SQSTM1/p62 protein levels following exercise, independent of exercise intensity, were in accordance with most studies [13,14,16,17]. Furthermore, the previously reported role of exercise intensity on p62 protein changes [15] may not be due to exercise intensity differences between protocols, but possibly related to other factors, such as total work performed. Interestingly, the only marker that showed a similar exercise-induced response across species and experimental conditions was the reduction in LC3B-II/I ratio, although through seemingly diverse mechanisms between rats and humans. Future studies should explore whether the exercise-induced decrease in LC3B-II/I ratio is reflective of increased autophagy flux.

Our data demonstrated that, following three weeks of high-volume HIIT in humans, there was an increase in basal LC3B-II protein level, suggesting an increase in autophagosome content (Figure 3). Our findings are in agreement with a recent study showing that four weeks of HIIT led to significant increases in LC3B-II protein content despite no significant increase in LC3B-I or LC3B-II/I ratio [19], and are in line with the results of a previous study where three weeks of one-legged knee extensor training led to an increase in LC3B-II protein levels [13]. However, others have not shown any effect of endurance training on LC3A/B-II protein levels, despite an increase in LC3A/B-I protein levels [17]. Whether training volume or intensity are more important for the training-induced changes in LC3B-II requires further research.

A limitation of human studies to date is the use of LC3B-II and SQSTM1/p62 protein levels to infer changes in autophagy flux. This has led to the idea that a decrease in LC3B-II protein levels following exercise could be reflective of a temporary decrease in autophagy flux [14]. In the present study, a protocol adapted from a rodent study [21] was used to perform a preliminary assessment of the effects of a single session of endurance exercise on ex vivo autophagy flux in human skeletal muscle. The results showed that autophagy flux (measured as net LC3B-II flux) did not decrease immediately after 0, 2.5, or even 24 h after exercise (Figure 4C). Although limited by the low number of participants, the effect size analyses suggested a moderate-to-large increase in ex vivo autophagy flux following exercise (+93–117%; ES = 0.79–0.88). Our findings in humans are in agreement with those from a rodent study showing a similar exercise-induced fold change in autophagy flux [10]. These preliminary findings would suggest there is a conserved exercise-induced increase in autophagy flux in rodents and humans (Figure 5B), despite a different exercise-induced LC3B protein regulation. The use of an ex vivo autophagy flux assay in future human studies will allow researchers to overcome the limitation of solely relying on static protein markers. Future research should interrogate the autophagy flux response to different stimuli (e.g., inactivity) in skeletal muscle and with larger sample sizes.

The main limitation of the present study is the low sample size in our ex vivo autophagy flux experiments. Nonetheless, our findings highlight the value of using this assay in human skeletal muscle studies. Furthermore, our rat study was limited to the use of soleus muscle in male Wistar rats and the autophagosome protein explored was limited to LC3B, whereas the role of other ATG8 family members in exercise and skeletal muscle autophagy remains unexplored. The use of the same mode of exercise (running vs. cycling), the addition of a control group, and the use of rats of similar age as in the human studies would have also strengthened the findings of the present study.

## 4. Materials and Methods

Four different studies were included in this manuscript: a single exercise session in rats (Study 1), exercise in humans at three different work-matched intensities above or below the maximal lactate steady state (MLSS) (Study 2), a 3-week high-volume high-intensity interval training in humans (Study 3), and a single exercise session in humans for the establishment of ex vivo autophagy flux (Study 4). All human participants were deemed healthy, and their characteristics can be found in Table 1. Studies were performed at Victoria University (Melbourne, Australia) and all analyses were performed under similar conditions in the same laboratory. All studies were approved by either the Victoria University Animal Ethics Committee (15/002) or the Victoria University Human Research Ethics Committee (HRE17-035; HRE15-126; HRE17-075). Informed consent was obtained from all human participants prior to study participation.

### 4.1. Study 1—Exercise in Rats

#### 4.1.1. Overview

A total of 28 male Wistar rats (8 weeks old) were obtained from the Animal Resource Centre (Perth, Australia). The Victoria University Animal Ethics Committee approved this study (AEC 15/002). All procedures were performed according to the Australian Code of Practice for the Care and Use of Animals for Scientific Purposes (National Health and Medical Research Council, Australia, 8th Edition). Rats were housed in groups of 2 to 4 in a temperature-controlled room and maintained with a chow diet (Specialty Feeds, Perth, WA) and water ad libitum on a 12:12 h light–dark cycle, 18–22 °C, with approximately 50% humidity. The animals underwent acclimation over three days, using five separate 15-min running sessions (ranging from being placed on a non-moving treadmill belt to running at a speed of 0.25 m·s^−1^). At least 48 h before the experimental exercise session, the animals performed an incremental exercise test. The incline of the treadmill was set at 10 degrees and the test was started at 0.16 m·s^−1^. The speed of the treadmill was increased 0.05 m·s^−1^ every three minutes. Animals were removed from the treadmill when they could no longer keep up with the speed despite encouragement (air puff).

#### 4.1.2. Experimental Session

Prior to the experimental day, rats had access to a chow diet until they were placed on the treadmill. Exercise was carried out three hours into their light phase (approximately 10 a.m.), which is associated with a minimal food intake period in rats [28]. Food was restricted after exercise to those animals that were exercised and samples collected 3 h post-exercise. Animals were exercised at 80% of their top speed achieved during the incremental test (approximately 0.38 m·s^−1^ at a 10 degree incline) for seven 2-min intervals interspersed with 1 min of rest. Rats were euthanised using 90 mg·kg^−1^ i.p. pentobarbitone prior to (REST; n = 9), immediately after (+0 h; n = 8), or 3 h (n = 11) after the completion of the exercise protocol, and the soleus was removed and immediately frozen in liquid nitrogen and stored at −80 °C (Figure 1A). The soleus muscle was chosen for analysis as it has been shown to have the highest levels of autophagy protein [5] and may better resemble the fibre type composition found in human skeletal muscles of moderately trained participants [29].

### 4.2. Study 2—Exercise in Humans: Effects of Exercise Intensity

#### 4.2.1. Overview

Ten healthy males volunteered for this study (Table 1). Participants were required to attend the laboratory at Victoria University 8 to 11 times. For the first trial, participants underwent a cycling graded exercise test (GXT) with 1-min increments, as previously described [30]. The following visits were dedicated to determining the maximal lactate steady state (MLSS), which was established by a series of 30-min constant power sessions. After the establishment of the MLSS, participants completed two constant power exercise sessions to exhaustion at +6% of the MLSS. Following this, they performed three experimental sessions that included skeletal muscle biopsies.

#### 4.2.2. Experimental Session

The three experimental sessions were performed in a randomised order at −18% (43.8 ± 12.1 min), −6% (38.1 ± 10.5 min), or +6% (33.8 ± 9.2 min) of the MLSS. The MLSS was selected as the reference point because it is a critical intensity that delineates heavy from severe exercise intensity [31], and three intensities (2 below and 1 above the MLSS) were chosen for the study. Participants were given 48 h of complete rest before each trial, and at least 7 days between the successive experimental trials. They were asked to maintain their normal diet and to replicate it on the day before and during the experimental trials. The experimental trials were performed in the morning following an overnight fast and participants were only allowed to drink water until the last biopsy of the present study. Biopsies were taken from the *vastus lateralis* muscle at rest before the start of exercise (REST), immediately upon completion of the exercise session (+0 h), and 3.5 h after the end of the exercise (+3.5 h) (Figure 2A). Samples were immediately cleaned of excess blood, fat, or connective tissue, and rapidly frozen in liquid nitrogen. Samples were stored at −80 °C until subsequent analyses.

### 4.3. Study 3—Exercise Training in Humans: Effect of High-Volume Training 

#### 4.3.1. Overview

Nine healthy male participants (Table 1) completed 20 days of twice-a-day high-intensity interval training (HIIT) as part of a larger study design, as previously published [32,33]. The exercise sessions consisted of 7 to 10 4-min intervals at a starting intensity of 50% of the power output between the lactate threshold and peak power in the GXT. Duration of the exercise sessions increased from 45 min to 60 min, and intensity was adjusted throughout this period. Skeletal muscle biopsies were obtained at rest before (PRE) and after (POST) the 20 days of high-volume HIIT (Figure 3A).

#### 4.3.2. Experimental Sessions

Participants were given 48 h of rest before the sample collection. All samples were obtained from the *vastus lateralis* muscle and participants were provided standardised meals, as previously described [32,34]. In brief, participants were provided with a standardised dinner (55 kJ kg^−1^ body mass (BM), providing 2.1 g carbohydrate (CHO) kg^−1^ BM, 0.3 g fat kg^−1^ BM, and 0.6 g protein kg^−1^ BM) and breakfast (41 kJ kg^−1^ BM, providing 1.8 g CHO kg^−1^ BM, 0.2 g fat kg^−1^ BM, and 0.3 g protein kg^−1^ BM) that were eaten at least 15 and 3 h prior to the muscle biopsy. Biopsies were taken at rest and were immediately cleaned of excess blood, fat, or connective tissue, and rapidly frozen in liquid nitrogen and stored at −80 °C for subsequent analyses.

### 4.4. Study 4—Exercise-Induced Autophagy Flux in Human Skeletal Muscle

#### 4.4.1. Overview

Samples from five healthy male participants from a larger unpublished study were analysed (Table 1). The number of participants included in this study was limited to those where sufficient skeletal muscle sample was obtained for the autophagy flux assay. The GXT protocol utilised in this study was the same as in Study 2. Participants had been familiarised with the exercise required as they had undertaken two GXTs and two exercise sessions in the two weeks prior to the experimental session.

#### 4.4.2. Experimental Session

Two participants underwent the following exercise: six 30-s ‘all-out’ cycling bouts against a resistance initially set at 0.075 kg·kg body mass^−1^ (~175% $\dot{W}$_max_), interspersed with a 4-min recovery period. The other three participants performed a session consisting of 90 min of continuous cycling at ~42% of $\dot{W}$_max_. Participants were given 72 h of rest before the experimental session. All samples were obtained from the *vastus lateralis* muscle and participants were provided standardised meals, as in previous studies [32,34]. In brief, participants were provided with a standardised dinner (55 kJ kg^−1^ body mass (BM), providing 2.1 g carbohydrate (CHO) kg^−1^ BM, 0.3 g fat kg^−1^ BM, and 0.6 g protein kg^−1^ BM) and breakfast (41 kJ kg^−1^ BM, providing 1.8 g CHO kg^−1^ BM, 0.2 g fat kg^−1^ BM, and 0.3 g protein kg^−1^ BM) that were eaten at least 15 and 3 h prior to the muscle biopsy. Biopsies were taken at rest before the start of exercise (REST), immediately upon completion of the exercise bout (+0 h), 2.5 h into the recovery (+2.5 h), and 24 h after the initial skeletal muscle sample (+24 h) (Figure 4A). Small muscle portions were immediately immersed into two separate vials with 3 mL of oxygenated DMEM, and the autophagy flux assay was started (see below in ex vivo autophagy flux assay). Once the protocol was finalised, samples were stored at −80 °C for subsequent analyses.

### 4.5. Skeletal Muscle Analyses

#### Preparation of Whole-Muscle Lysates

Approximately 10 to 20 mg of frozen muscle was homogenised two times for two minutes at a speed of 30 Hz with a TissueLyser instrument (Qiagen, Canada) in an ice-cold lysis buffer (1:20 *w*/*v*) containing 50 mM Tris-HCl, 150 mM NaCl, 1 mM EDTA, 5 mM Na_4_P_2_O_7_, 1 mM Na_3_VO_4_, and 1% NP-40, with added protease and phosphatase inhibitors at a 1:100 concentration (Cell Signaling Technology). Protein concentration was determined using a commercial colourimetric assay (Bio-Rad Protein Assay kit II, Bio-Rad Laboratories Pty Ltd., Gladesville, NSW, Australia) and lysates were then diluted with an equal volume in 2× Laemmli buffer containing 10% B-mercaptoethanol.

### 4.6. Western Blotting

For each protein of interest, a signal linearity test was conducted to determine the ideal loading amount. Muscle lysates were then loaded in equal amounts (10 to 20 μg) and separated by electrophoresis for 1.5 to 2.5 h at 100 V using precast stain-free SDS-PAGE gels (4–20%). Once resolved, the gels were wet transferred onto LF PVDF membranes using a Turbo Transfer system (Bio-rad Laboratories Pty Ltd., Gladesville, NSW, Australia). Membranes were blocked at room temperature for 1 h using 5% skim milk or 5% bovine serum albumin (BSA) in Tris-buffered saline (TBS) 0.1% tween-20 (TBS-T). After 3 × 5-min washes in TBS-T, membranes were incubated overnight at 4 °C with gentle agitation in primary antibody solutions (1:1000 antibody in 5% BSA, plus 0.02% Na Azide). The antibody for LC3B was purchased from Cell Signalling (#3868S) and the antibody for SQSTM1/p62 from Abcam (#ab56416). The following morning, membranes were washed 3 × 5 min in TBS-T and subsequently incubated under gentle agitation at room temperature with the appropriate host species-specific secondary antibody for 60 to 90 min in 1–5% skim milk in TBS-T. Membranes were washed again for 3 × 5 min in TBS-T before being immersed for 5 min under gentle agitation at room temperature in Clarity ECL detection substrate (Bio-rad Laboratories Pty Ltd., Gladesville, NSW, Australia). Protein bands were visualised using a Bio-Rad ChemiDoc imaging system and band densities were determined using Bio-Rad ImageLab software (Bio-Rad Laboratories Pty Ltd., Gladesville, NSW, Australia). All samples for each participant were loaded on the same gel, along with different concentrations of a mixed-homogenate internal standard (IS), and a calibration curve plotted of density against protein amount. From the subsequent linear regression equation, protein abundance was calculated from the measured band intensity for each lane on the gel. Total protein content of each lane was obtained from the stain-free image of the membrane and was used for normalisation of the results.

### 4.7. RNA Extraction and Reverse Transcription

Frozen muscle (10 to 15 mg) was used to isolate RNA using the RNeasy Mini Kit (Qiagen, Canada) according to the manufacturer’s instructions. Samples were homogenised using the TissueLyser II (Qiagen, Canada). RNA concentration and purity was determined spectrophotometrically (NanoDrop 2000, Thermo Fisher Scientific, Wilmington, DE). For each sample, 1 μg of RNA was transcribed into cDNA on a thermal cycler (S1000TM Thermal Cycler, Bio-Rad, USA) using the iScriptTM cDNA Synthesis Kit (Bio-Rad, USA) and the following incubation profile: 5 min at 25 °C, 30 min at 42 °C, and 5 min at 85 °C. The transcription was performed with random hexamers and oligo dTs in accordance with the manufacturer’s instructions. A reverse transcriptase (RT)-negative control was also generated. cDNA was stored at −20°C until subsequent analysis.

### 4.8. qPCR

All samples were run together to decrease technical variation and were performed following previously published guidelines [35]. Forward and reverse primers for the target and housekeeping genes were designed based on NCBI RefSeq using NCBI Primer-BLAST (www.ncbi.nlm.nih.gov/BLAST/ (accessed on 1 October 2021)) and are shown in Table 2. Specificity of the amplified product was confirmed by melting point dissociation curves. The mRNA expression was performed by quantitative real-time RT-PCR (Mastercycler^®^ RealPlex2, Eppendorf, Germany) using a 5 μL PCR reaction volume with SYBR Green supermix (SsoAdvanced™ Universal SYBR^®^ Green Supermix, Bio-Rad, USA). All samples were run in duplicate simultaneously with template-free controls using an automated pipetting system (epMotion 5070, Eppendorf, Germany). The following PCR cycling patterns were used: initial denaturation at 95° C (3 min), 40 cycles of 95° C (15 s) and 60° C (60 s). For study 1, cyclophilin (*Ppia*), beta-2-microglobulin (*B2m*), and Actin Beta (*Actb*) were used as housekeeping genes. For study 2, TATA-Box binding protein (*TBP*), glyceraldehyde 3-phosphate dehydrogenase (*GAPDH*), and B2M were used as housekeeping genes. Expression of each target gene was calculated as 2 to the power of the negative difference between the geometric mean of the cycle threshold (CT) of the housekeeping genes and the CT of the target gene, and expressed as 2^−^^Δ^^CT^.

### 4.9. Ex Vivo Autophagy Flux Assay

The following protocol was adapted from previous studies performing ex vivo autophagy flux in rodents [21,36]. Upon collection of the skeletal muscle sample, two small pieces (~20 mg all together) were placed in 3 mL of oxygenated DMEM CO_2_ independent media (ThermoFisher #18045088) at 37 °C. The tissues were then incubated with continuous oxygenation for 1 h with (‘treated’ sample, with inhibitors) or without (‘untreated’ sample) 60 µL of NH_4_Cl (20 µL·mL^−1^; 40 mM; Merck #101142) and 30 µL Leupeptin (10 µL·mL^−1^; 100 uM; Sigma Aldrich #L2884). Upon completion of a 1-h incubation, samples were snap-frozen and stored at −80 °C until further analysis. Autophagy flux (Net LC3B-II flux) was obtained by subtraction of the densitometric value of LC3B-II from treated compared to the untreated sample. 

### 4.10. Statistical Analysis

All values are reported as mean ± standard deviation (SD). All statistical analyses were carried out on the raw values normalised to the total protein loading and calibration curve. For Study 1, one-way repeated measures of ANOVA with Holm-Sidak post hoc were used. For Study 2, two-way repeated measures of ANOVA were used, and main effects and interactions were further analysed using Holm-Sidak post hoc tests. For Study 3, a two-tailed paired Student’s t-test was used. For Study 4, a one-way ANOVA with Holm-Sidak post hoc tests was utilised. Data distribution was assessed using Shapiro–Wilk test and, when non-normally distributed, the normality of the data was achieved by log-transformation. Effect sizes (ES) were quantified and defined as: small (0.2), moderate (0.5), large (0.8), and very large (1.3). Statistical significance was set at *p* < 0.05 for all analyses. GraphPad Prism 8.3 software was used for the statistical analysis. 

## 5. Conclusions

In conclusion, the results of the current study showed that exercise-induced LC3B protein changes differ between rats and humans. This indicates caution must be taken when extrapolating autophagy protein results from rodents to humans. Furthermore, findings from the current study show that a reduction in LC3B-II protein levels following exercise in humans was consistent across exercise intensities but was not indicative of a decrease in autophagy flux. This suggests that studies should avoid looking at ‘static’ levels of LC3 protein levels and include autophagy flux assays to provide a more valid assessment of dynamic changes in autophagy with exercise.

## Figures and Tables

**Table 1:** Descriptive data of the human participants recruited for studies 2, 3, and 4. Data are mean ± SD. $\dot{V}$O_2peak_ = peak of oxygen uptake; $\dot{W}$_max_ = maximal aerobic power; MLSS = maximal lactate steady state; SIE = sprint interval exercise; MICE = moderate-intensity continuous exercise.

	Age (Year)	$\dot{V}$O_2peak_ (mL·min^−1^·kg^−1^)	Trial	Relative Exercise Intensity (% $\dot{W}$_max_)	Absolute Exercise Intensity (W)
Study 2 (n = 10)	27.5 ± 7.7	55.8 ± 10.0	−18% MLSS	51 ± 4	181 ± 39
			−6% MLSS	59 ± 4	207 ± 45
			+6% MLSS	67 ± 5	234 ± 51
Study 3 (n = 9)	22.4 ± 5.2	47.0 ± 7.5	-		
Study 4 (n = 5)	30.0 ± 7.3	48.1 ± 4.4	SIE MICE	175 ± 21 42 ± 2	444 ±179 142 ± 35

**Table 2:** qPCR gene primers used.

Species	Gene	Sequence
Rat	*Ppia*	F-TCTGCACTGCCAAGACTGAG R-GTCCACAGTCGGAGATGGTG
Rat	*B2m*	F-ACCCACCGAGACCGATGTA R-GGTCCCAGGTGACGGTTTT
Rat	*Actb*	F-CGATATCGCTGCGCTCGT R-ATACCCACCATCACACCCTG
Rat	*Map1lc3b*	F-CGCCGGAGCTTCGAACAA R-TCACTGGGATCTTGGTGGGG
Rat	*Sqstsm1/p62*	F-GCGCACTACCGCGATGA R-ACATGGCCATTGTCAGTTCCT
Human	*TBP*	F-CAGTGACCCAGCAGCATCACT R-AGGCCAAGCCCTGAGCGTAA
Human	*GAPDH*	F-AATCCCATCACCATCTTCCA R-TGGACTCCACGACGTACTCA
Human	*B2M*	F-TGCTGTCTCCATGTTTGATGTATCT R-TCTCTGCTCCCCACCTCTAAGT
Human	*MAP1LC3B*	F-CAGCATCCAACCAAAATCCCG R-TTGAGCTGTAAGCGCCTTCTAA
Human	*SQSTM1/p62*	F-AGAATCAGCTTCTGGTCCATCGG R-CTTTTCCCTCCGTGCTCCAC

## Abbreviations

LC3, microtubule-associated proteins 1A/1B light chain 3; p62, ubiquitin-binding protein p62; INH, inhibitors; MLSS, maximal lactate steady state; HIIE, high-intensity interval exercise; HIIT, high-intensity interval training; MICE, moderate-intensity continuous exercise; SIE, sprint-interval exercise; GXT, graded exercise test;$\dot{V}$O_2peak_, peak oxygen uptake; $\dot{V}$O_2max_, maximal oxygen uptake; W_LT_, power at the lactate threshold; $\dot{W}$_max_, maximal aerobic power.
